# Prognostic Utility of dNLR, ALRI, APRI, and SII in COVID-19 Patients with Diabetes: A Cross-Sectional Study

**DOI:** 10.3390/diagnostics14151685

**Published:** 2024-08-04

**Authors:** Alexandra Ioana Danila, Flavius Cioca, Sai Teja Gadde, Sai Praveen Daruvuri, Romulus Timar, Elena Hogea

**Affiliations:** 1 Doctoral School, “Victor Babes” University of Medicine and Pharmacy, Eftimie Murgu Square 2, 300041 Timisoara, Romania; alexandra.danila@umft.ro; 2 Department of Anatomy and Embryology, “Victor Babes” University of Medicine and Pharmacy, Eftimie Murgu Square 2, 300041 Timisoara, Romania; 3 Discipline of Medical Statistics and Bioinformatics, “Victor Babes” University of Medicine and Pharmacy, Eftimie Murgu Square 2, 300041 Timisoara, Romania; 4 Faculty of General Medicine, All India Institute of Medical Sciences (AIIMS), Mangalagiri 522503, India; gaddesai0626@gmail.com; 5 Faculty of General Medicine, Bukovinian State Medical University, Teatralna Square, 2, 58002 Chernivtsi, Ukraine; praveenzzz39@gmail.com; 6 Department of Internal Medicine II, Division of Diabetes, Nutrition and Metabolic Diseases, “Victor Babes” University of Medicine and Pharmacy, Eftimie Murgu Square 2, 300041 Timisoara, Romania; timar.romulus@umft.ro; 7 Discipline of Microbiology, “Victor Babes” University of Medicine and Pharmacy Timisoara, 300041 Timisoara, Romania; hogea.elena@umft.ro

**Keywords:** diabetes mellitus, COVID-19, SARS-CoV-2

## Abstract

The coronavirus disease 2019 (COVID-19) pandemic has necessitated the identification of biomarkers that can predict disease severity, particularly in vulnerable populations such as individuals with diabetes. This study aims to evaluate the predictive value of inflammatory and liver function markers, specifically derived Neutrophil to Lymphocyte Ratio (dNLR), aspartate aminotransferase (AST)-to-lymphocyte ratio (ALRI), AST to Platelet Ratio Index (APRI), and Systemic Inflammation Index (SII), in COVID-19 patients with and without diabetes. This cross-sectional study included 336 participants, comprising 168 patients with diabetes matched with 168 without, based on gender, body mass index (BMI), and COVID-19 severity at hospitalization. The study was conducted at Victor Babes Hospital for Infectious Diseases and Pulmonology from January 2021 to December 2023. All participants had a confirmed SARS-CoV-2 infection and met the inclusion criteria of being 18 years or older with type 1 or type 2 diabetes as per American Diabetes Association guidelines. At 3 days post symptom onset, significant differences in inflammatory and liver function markers were observed between the two groups. The dNLR, ALRI, APRI, and SII were notably higher in diabetic patients. At a dNLR cutoff of 2.685, the sensitivity and specificity were 70.312% and 65.978%, respectively, with an AUC of 0.624 (*p* < 0.001). The ALRI showed a cutoff of 0.812, with a sensitivity of 76.429% and specificity of 69.541% (AUC 0.752, *p* < 0.001). These markers demonstrated statistically significant hazard ratios at both 3 and 7 days, indicating their predictive relevance for severe COVID-19 outcomes. For instance, at 7 days, SII demonstrated a hazard ratio of 2.62 (CI: 1.29–5.04, *p* < 0.001), highlighting its strong prognostic capability. The study successfully identified significant differences in inflammatory and liver function markers between COVID-19 patients with and without diabetes, with these markers showing good predictive value for disease severity. The results underscore the potential of these biomarkers, particularly ALRI and SII, as valuable tools in managing COVID-19, aiding in the timely identification of patients at increased risk of severe outcomes.

## 1. Introduction

As of the latest updates, COVID-19 continues to affect global populations significantly [1]. As of March 2024, over 774 million confirmed cases and more than seven million deaths have been reported globally [2]. Notably, there has been a decreasing trend in new cases and fatalities in recent months. For instance, between early February and late March 2024, the number of new cases and deaths fell by 35% and 64%, respectively [3].

Diabetes significantly increases the risks associated with COVID-19, leading to heightened rates of complications and mortality. This association is supported by empirical evidence [4,5,6] that diabetic patients, due to their deficient metabolic and immune systems, higher oxidative stress and pro-inflammatory proteins, are more susceptible to severe infections [7,8,9,10,11]. This vulnerability necessitates specialized care and targeted research to address the complex management challenges these patients face, particularly when infected with COVID-19. Such specialized approaches are critical for improving outcomes and are discussed in detail in the literature regarding diabetic patients [12,13,14], emphasizing the need for a deeper understanding of the interplay between chronic conditions like diabetes and infectious diseases such as COVID-19.

In order to emphasize the higher risk of severity and mortality among diabetic patients, clinical and paraclinical particularities are essential to identify at admission and during hospitalization. As such, inflammatory markers have shown considerable promise in predicting COVID-19 outcomes. Neutrophil to Lymphocyte Ratio (NLR), Platelet to Lymphocyte Ratio (PLR), Aspartate Aminotransferase (AST) to Platelet Ratio Index (APRI), aspartate aminotransferase-to- lymphocyte ratio (ALRI), and the Systemic Immune-Inflammation Index (SII) are among the indicators used to assess the severity and progression of infections and were proved by different studies to show superiority in predicting COVID-19 outcomes than the commonly used laboratory biomarkers [15,16,17,18,19].

Liver function tests are another essential aspect of managing COVID-19, especially given the systemic impact of the SARS-CoV-2 virus beyond the respiratory tract, and its affinity for ACE2 [20,21]. Therefore, the hypothesis of this study posits that inflammatory and liver function markers (dNLR, ALRI, APRI, and SII) are significantly higher in SARS-CoV-2-infected patients with diabetes compared to those without diabetes, and that these differences can predict disease severity. The primary objective is to evaluate the predictive value of inflammatory markers for COVID-19 severity in diabetic patients with SARS-CoV-2 infection.

## 2. Materials and Methods

### 2.1. Study Design and Ethics

This cross-sectional study focused on patients with diabetes mellitus admitted to the Victor Babes Hospital for Infectious Diseases and Pulmonology from January 2021 to December 2023 for SARS-CoV-2 infection. Data collection involves both electronic health records and manual chart reviews of the hospital. The study protocol has been approved by the institutional review board of each participating center, ensuring adherence to ethical standards. All patient data were anonymized prior to analysis to maintain confidentiality. This research complies with the ethical guidelines of the Declaration of Helsinki, EU Good Clinical Practice Directives (2005/28/EC), and International Council for Harmonization of Technical Requirements for Pharmaceuticals for Human Use (ICH) guidelines and has received approval from the Local Commission of Ethics for Scientific Research.

### 2.2. Inclusion and Exclusion Criteria

The study included participants who had a confirmed diagnosis of type 1 or type 2 diabetes based on the American Diabetes Association guidelines [22]. Additionally, all participants were required to have a laboratory-confirmed SARS-CoV-2 infection as verified by RT-PCR testing. The study was restricted to adult participants, with all individuals being aged 18 years or older at the time of their COVID-19 diagnosis. Consent for the analysis of personal medical records was mandatory for inclusion. The study required participants to have available laboratory and biological parameters essential for calculating the prognostic scores being studied, including dNLR, ALRI, APRI, and SII.

Exclusion criteria comprised the following: individuals diagnosed with gestational diabetes or other unspecified forms of diabetes were not included in the study. Participants who exhibited high serum glucose levels without a definitive diagnosis of type 1 or type 2 diabetes were also excluded. The study further excluded any participants with incomplete medical records, particularly those lacking critical data such as HbA1c levels, detailed COVID-19 treatment information, or outcome data. Lastly, individuals who did not provide consent for their medical records to be used in the study were excluded.

### 2.3. Data Collection and Variables

The variables included demographic information such as age and gender, as well as specific diabetes-related data like the type of diabetes (type 1 or type 2), duration of the disease, and HbA1c levels. The severity of COVID-19 in the participants was classified according to the World Health Organization criteria [23], encompassing mild, moderate, and severe/critical categories based on symptoms, oxygen requirements, and chest imaging findings. We gathered details on hospitalization, including ICU admissions and whether patients required mechanical ventilation. The Charlson Comorbidity Index (CCI) was used to quantify the burden of comorbidities that might influence the risk of death from COVID-19. We calculated the inflammatory scores, namely dNLR, ALRI, APRI, and SII. Patients were matched by COVID-19 severity. Laboratory data and inflammatory scores were calculated at 3 days and 7 days after the onset of first COVID-19 symptoms. An additional control group consisting of patients with diabetes and without COVID-19 was included in the analysis as comparison to the group of COVID-19 patients with diabetes in order to determine if changes in inflammatory markers were due to COVID-19 or diabetes alone.

### 2.4. Statistics

The data were managed and analyzed using R statistical software (version 4.0.3). Continuous variables were presented as means ± standard deviation (SD) and categorical variables as frequencies and percentages. The Student’s *t*-test was used for comparing continuous variables between diabetic and non-diabetic groups, while the Chi-square test was used for categorical data. To assess the predictive value of inflammatory and liver function markers on COVID-19 severity, receiver operating characteristic (ROC) curves were generated to determine optimal cutoff values, with calculation of sensitivity, specificity, and the Area Under the Curve (AUC). A Cox proportional hazards regression model was applied to estimate hazard ratios (HRs) for severe outcomes of COVID-19 based on marker levels exceeding their determined cutoffs. The model was adjusted for potential confounders including age, gender, BMI, and the Charlson Comorbidity Index (CCI). Before applying the Cox model, we tested the proportional hazards assumption using Schoenfeld residuals. Multivariable logistic regression was also conducted to adjust for other influential factors such as underlying diseases, type of diabetes, and duration of disease, to provide a comprehensive analysis of the factors impacting COVID-19 severity. Statistical significance was set at a *p*-value of less than 0.05.

In this study, the reference group for the dependent variable, severity of COVID-19, consisted of patients categorized as having mild symptoms. This group served as the baseline to which moderate and severe outcomes were compared. The hazard ratios (HRs) provided in the analysis represent the risk of severe outcomes relative to this mild reference group. Adjustments in the models were made to account for potential confounders identified in the reviewer’s comments, such as underlying diseases and demographic variables (age, gender), as well as the type and duration of diabetes, which could influence the severity of COVID-19.

## 3. Results

A total of 168 patients without diabetes were matched with 168 patients with diabetes based on their COVID-19 severity. Age-wise, the average for patients without diabetes was 50.5 years with a standard deviation (SD) of 12.3, while those with diabetes had an average age of 52.8 years with an SD of 11.7; this difference was not statistically significant (*p*-value = 0.080). Gender distribution showed a slightly higher percentage of men in the diabetes group (58.33%) compared to the no diabetes group (54.76%). Body mass index (BMI) averages were 26.8 (SD = 4.2) for the no diabetes group and 27.3 (SD = 5.1) for the diabetes group, showing no significant difference (*p*-value = 0.327). Regarding diabetes types among patients in the diabetes group, 17.86% had type 1 diabetes mellitus (T1DM) and 82.14% had type 2 diabetes mellitus (T2DM). Vaccination rates against COVID-19 were higher in the diabetes group (89.29%) compared to the no diabetes group (84.52%), but this difference was not significant (*p*-value = 0.204).

Significantly more patients with diabetes had a Charlson Comorbidity Index (CCI) greater than 2 (44.64%) compared to those without diabetes (29.76%), indicating a higher burden of comorbid conditions in the diabetes group (*p*-value = 0.005). No significant differences were observed in COVID-19 severity distribution between the groups (*p*-value = 0.429), considering the case-matching of the studied patients. However, there were significant differences in the rates of ICU admissions, mechanical ventilation, and mortality, all of which were higher among patients with diabetes. Specifically, ICU admissions were reported in 15.48% of the diabetes group compared to 7.14% in the no diabetes group (*p*-value = 0.016), mechanical ventilation was needed for 11.31% of patients with diabetes versus 3.57% of those without (*p*-value = 0.007), and mortality was 7.14% in the diabetes group compared to 2.38% in the no diabetes group (*p*-value = 0.040), as presented in Table 1.

At 3 days from symptom onset blood glucose levels were markedly higher in patients with diabetes (159.22 ± 49.73 mg/dL) compared to those without diabetes (97.36 ± 14.28 mg/dL), with a highly significant *p*-value of less than 0.001, underscoring the expected impact of diabetes on glucose metabolism. Similarly, C-reactive protein was significantly elevated in diabetic patients (69.97 ± 49.86 mg/L) compared to non-diabetic patients (39.64 ± 34.15 mg/L), with a *p*-value of 0.002. Additionally, D-dimer levels were higher in the diabetes group (0.68 ± 0.39 μg/mL) than in the non-diabetes group (0.48 ± 0.32 μg/mL), with a *p*-value of 0.014. Creatinine levels also differed significantly between the groups, being higher in the diabetes group (1.34 ± 0.36 mg/dL) compared to the non-diabetes group (0.97 ± 0.22 mg/dL), with a *p*-value of 0.003.

Lymphocyte counts were lower in patients with diabetes (1.18 ± 0.43 × 10^9^/L) versus those without (1.52 ± 0.46 × 10^9^/L), which is significant with a *p*-value of 0.01. Although the differences in platelet count and white blood cell count were analyzed, they did not show statistically significant differences between the two groups. The liver enzymes AST and ALT showed significant elevations in diabetic patients compared to non-diabetics (Table 2).

Blood glucose levels significantly differed between the groups, with diabetic patients showing much higher levels (149.53 ± 39.86 mg/dL) compared to those without diabetes (95.42 ± 10.67 mg/dL), with a *p*-value of less than 0.001. C-reactive protein was also significantly elevated in the diabetes group (54.32 ± 30.47 mg/L) compared to the non-diabetes group (31.74 ± 19.83 mg/L), with a *p*-value of less than 0.001. The D-dimer levels were higher in diabetic patients (0.63 ± 0.28 μg/mL) than in those without diabetes (0.42 ± 0.21 μg/mL), with a *p*-value of 0.006.

Creatinine levels were also significantly higher in the diabetes group (1.27 ± 0.28 mg/dL) compared to the non-diabetes group (0.91 ± 0.19 mg/dL), with a *p*-value of 0.001. Interestingly, lymphocyte counts were significantly higher in diabetic patients (4.38 ± 0.48 × 10^9^/L) compared to non-diabetic patients (3.83 ± 0.45 × 10^9^/L), with a *p*-value of less than 0.001. Platelet counts were lower in patients with diabetes (202.14 ± 45.67) compared to those without (231.78 ± 40.35), with a *p*-value of 0.033. White blood cell counts were higher in diabetic patients (6.79 ± 1.69 × 10^9^/L) compared to non-diabetics (6.01 ± 1.04 × 10^9^/L), with a *p*-value of less than 0.001, further supporting the presence of an elevated inflammatory state. Both liver enzymes, AST and ALT, showed significant elevations in diabetic patients, with *p*-values of less than 0.001, indicating a higher degree of hepatic stress or inflammation in these patients. Laboratory scores including dNLR, ALRI, APRI, and SII were all significantly higher in the diabetes group, with *p*-values ranging from 0.002 to less than 0.001 (Table 3).

At 3 days from symptom onset, the dNLR had a cutoff value of 2.685, demonstrating a sensitivity of 70.312% and a specificity of 65.978%, with an AUC of 0.624 (*p*-value < 0.001). The ALRI presented a cutoff value of 0.812, with higher sensitivity and specificity (76.429% and 69.541%, respectively) and an AUC of 0.752, indicating a good predictive value with statistical significance (*p* < 0.001). The APRI had a cutoff of 0.965, yielding lower sensitivity and specificity (62.837% and 67.395%, respectively) compared to ALRI, with an AUC of 0.599 and a *p*-value of 0.006, suggesting it is less effective than ALRI but still statistically significant. Also, the SII showed a cutoff of 645.285, with sensitivity and specificity of 74.158% and 68.264%, respectively, and an AUC of 0.768 (*p* < 0.001).

At 7 days from symptom onset, the dNLR had a slightly lower cutoff value of 2.539 compared to the 3-day mark, with improved sensitivity and specificity (72.105% and 67.321%, respectively) and an AUC of 0.652, maintaining statistical significance (*p* < 0.001). The ALRI’s cutoff increased to 0.892, with sensitivity and specificity slightly decreasing to 74.991% and 70.002%, respectively, but the AUC increased to 0.756 (*p* < 0.001), suggesting consistent prognostic utility over time. The APRI’s cutoff rose to 1.101, showing better sensitivity and specificity (69.483% and 73.51%, respectively) compared to its 3-day measurement, with an AUC of 0.706 (*p* < 0.001). Lastly, the SII’s cutoff at 7 days was 681.742, the highest of all parameters, with sensitivity of 75.869% and specificity of 69.415%, and the highest AUC of 0.781 (*p* < 0.001), marking it as a particularly effective prognostic marker (Table 4).

At 3 days from symptom onset, the dNLR showed a hazard ratio of 1.32, indicating that patients with values above the cutoff had a 32% higher risk of developing severe COVID-19 compared to those below the cutoff. The ALRI demonstrated a higher hazard ratio of 1.48 (CI: 1.12–1.97), with a *p*-value of 0.006, suggesting a 48% increased risk of severe disease among those above the cutoff, indicating a stronger predictive power for ALRI at this early stage. The APRI, however, was not a significant predictor. The SII showed a significantly higher hazard ratio of 1.53 (CI: 1.20–3.92), with a *p*-value of 0.001, indicating that those above the cutoff had a 53% increased risk of severe disease, making it a strong prognostic marker at this timeframe.

**Figure 1:**
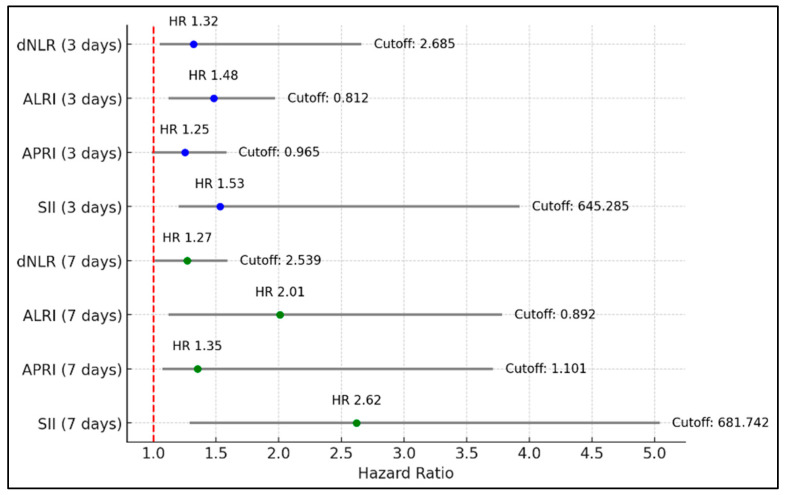
Regression analysis for severe COVID-19 development in patients with diabetes.

At 7 days from symptom onset, the dNLR’s hazard ratio was slightly reduced to 1.27 (CI: 1.01–1.59) with a *p*-value of 0.042. ALRI at 7 days exhibited a higher hazard ratio of 2.01 (CI: 1.12–3.78), with a *p*-value of 0.003, suggesting a doubled risk for severe COVID-19, indicating its increasing relevance as the disease progresses. The hazard ratio for APRI increased to 1.35 (CI: 1.07–3.71) with a *p*-value of 0.012. Finally, the SII showed the highest hazard ratio of 2.62 (CI: 1.29–5.04) with a *p*-value of less than 0.001, indicating a significantly higher risk of severe disease in patients exceeding this threshold, underscoring its utility in late-stage disease prognosis, as presented in Table 5 and Figure 1.

The adjusted analyses revealed significant relationships between several biomarkers and the severity of COVID-19, particularly after adjusting for confounders such as age, BMI, gender, and the presence of comorbidities measured by the Charlson Comorbidity Index (CCI). The Systemic Inflammation Index (SII) and ALRI showed strong associations with increased severity of COVID-19, suggesting these markers could be effective in predicting worse outcomes in patients with diabetes. Adjustments for underlying diseases and the type of diabetes (type 1 vs. type 2) also influenced the hazard ratios, highlighting the importance of considering these factors in the analysis to avoid biased conclusions. The presence of diabetes itself, particularly as it progresses in duration, modestly increased the risk of severe COVID-19, underscoring the multifactorial impact of chronic conditions on infectious disease outcomes, as presented in Table 6.

## 4. Discussion

### 4.1. Analysis of Findings

In the context of COVID-19, particularly among patients with diabetes, studying inflammatory markers and liver function is crucial due to the unique challenges posed by the interaction of these chronic and acute condition; this can provide insights into the severity of the immune response, which is often exacerbated in diabetic patients. Similarly, liver function tests help assess the impact of COVID-19 on metabolic processes, which are frequently disturbed in diabetes, on top of the already demonstrated affinity of SARS-CoV-2 on the ACE2 present in the liver. Understanding these dynamics is vital for tailoring treatment strategies to reduce the severity and mortality associated with COVID-19 in this vulnerable population.

The study’s significant findings reveal insights into the role of inflammatory and liver function markers in predicting COVID-19 severity among patients with diabetes. Particularly, markers such as the dNLR, ALRI, APRI, and SII showed distinct prognostic significance. The SII, with its highest AUC value across both time frames (3 and 7 days post symptom onset), underscores its utility as an effective prognostic marker. Significantly, based on the hazard ratios, the ALRI showed an increasing relevance with a hazard ratio that nearly doubled from the third to the seventh day post symptom onset, suggesting its potential role in reflecting worsening conditions. Conversely, the APRI’s lower sensitivity and specificity suggest it may be less reliable as a prognostic tool compared to ALRI and SII, although its predictive relevance increases over time.

The study’s findings reflect similarities and variances observed in existing literature on diabetes, hyperglycemia, and COVID-19 severity. Specifically, Gharaibeh et al. [24] identified increased COVID-19 severity in diabetic patients, evidenced by statistically significant elevations in inflammatory markers such as CRP and white blood cells. This suggests that diabetic patients tend to exhibit an exaggerated inflammatory response, which is associated with more severe disease manifestations. In contrast, Geetha et al. [25] investigated new-onset hyperglycemia in non-diabetic patients and found no statistically significant relationship between most inflammatory markers (CRP, ferritin, D-dimer) and hyperglycemia, aside from a minor correlation with LDH levels. Despite the minimal linkage to inflammation, their study noted significantly higher mortality rates (24.2% vs. 9.1%) and longer hospital stays (8.89 vs. 6.69 days) among hyperglycemic patients, highlighting its adverse impact on COVID-19 outcomes. These differing outcomes could be attributed to variations in patient populations, hyperglycemia definitions, or the inflammatory markers studied, underscoring the complex interplay between metabolic disturbances and COVID-19 severity.

The study by Karaaslan et al. [26] demonstrated a significant correlation between SII and mortality among 191 COVID-19 patients, where a higher SII (cut-off value > 618.8) was associated with a 4.68-fold increase in mortality, underlining SII’s predictive accuracy with an area under the curve of 0.751, sensitivity of 80.0%, and specificity of 61.5%. This study also highlighted the strong correlations of SII with other inflammation markers such as the neutrophil-to-lymphocyte ratio and platelet-to-lymphocyte ratio, with AUC values of 0.812 and 0.841, respectively, which were higher than in our study. Conversely, the review by Karimi et al. [27] acknowledged the effectiveness of various systemic inflammation markers like NLR, SII, and CRP ratios in assessing COVID-19 prognosis but pointed out that despite the promising results, NLR remains the most validated marker due to extensive studies confirming its prognostic value, although other markers including SII showed potential in certain studies. This evidence underscores the value of integrating SII measurements, specifically over values higher than 600, as found by our study and Karaaslan’s study, along with other established markers like NLR to enhance the accuracy and efficacy of prognosis in clinical settings, potentially improving patient outcomes through more precise risk stratification.

Other similar studies emphasize the predictive value of systemic inflammatory markers in assessing COVID-19 severity and mortality. Mangoni and Zinellu’s meta-analysis on the Systemic Inflammation Index showed significantly higher SII values in patients with severe disease or non-survivor status (standard mean difference = 0.91, *p* < 0.001), with an area under the curve of 0.77, highlighting its role in early risk stratification [28]. Similarly, Dymicka-Piekarska’s findings reveal that the neutrophil-to-lymphocyte ratio significantly correlates with disease severity, evidenced by an odds ratio of 1.050 (*p* = 0.002) for higher mortality risks [29]. Therefore, these studies suggest that the SII is highly effective for early risk stratification in severe COVID-19 cases due to its strong predictive accuracy, while the NLR, with its specific correlation to mortality, is particularly useful for assessing risk in settings where a single blood parameter is preferable for swift decision-making.

Yılmaz et al. [30] explored the associations between mortality and several biomarkers in 466 critically ill COVID-19 patients, revealing a significant predictive value of the dNLR and the Acute Physiology and Chronic Health Evaluation II (APACHE II) scores. They documented that patients in the non-survival group exhibited significantly higher levels of leukocytes, neutrophils, dNLR, and higher APACHE II scores, with the dNLR showing a notable cut-off value of 3.64 for predicting 28-day mortality (*p* = 0.002). Conversely, Zhao et al. [31] evaluated inflammatory markers in 285 COVID-19 patients, comparing them with 446 influenza A patients. Their findings underscored that severe COVID-19 cases had markedly elevated levels of NLR, Systemic Inflammation Index, and interleukin-6 (IL-6), among others, with NLR presenting an area under the curve of 0.76, indicating its efficacy in distinguishing between mild and severe cases.

In the retrospective observational study by Citu et al. [32], inflammatory biomarkers were evaluated for their predictive value in COVID-19 mortality among 108 hospitalized patients. They found that the NLR, dNLR, and MLR were significant predictors of mortality. Specifically, the study reported elevated NLR and dNLR as independent predictors with ORs of 4.14 and 14.09, respectively, highlighting dNLR’s strong association with poor clinical outcomes. The median age of the patients was 63.31 years, and the in-hospital death rate was 15.7%. In contrast, the prospective cohort study by Chelariu et al. [33] included 607 COVID-19 patients, assessing hematological indices derived from complete blood counts for predicting the need for ICU admission and short-term mortality. Elevated NLR scores followed up after a median of 8 days were predictive of increased ICU admissions (OR: 1.14) and short-term mortality (OR: 1.30). MLR similarly showed a significant predictive value for ICU admissions and mortality, indicating a 2.5-fold and 2.2-fold increase in odds, respectively. Both studies, along with our study findings, underscore the importance of CBC-derived inflammatory markers like NLR and MLR in predicting adverse outcomes in COVID-19 patients, thereby aiding in timely and effective clinical decision-making.

The current study also highlighted the higher rates of ICU admissions, mechanical ventilation, and mortality among diabetic patients, suggesting that diabetes significantly exacerbates the severity of COVID-19. This correlation underscores the importance of closely monitoring inflammatory and liver function markers in diabetic patients as part of COVID-19 severity assessments. This study uniquely contributes to existing knowledge by evaluating the predictive value of dynamic changes in inflammatory and liver function markers in diabetic patients at high risk for severe COVID-19. It employs a novel approach by measuring inflammatory scores, including dNLR, ALRI, APRI, and SII at two critical time points—3 and 7 days post-diagnosis. This time-based analysis allows for an in-depth understanding of the progression of the inflammatory response and its correlation with disease severity in this vulnerable population, highlighting a strategic period for potential therapeutic intervention and improved clinical outcomes.

### 4.2. Study Limitations and Future Perspectives

Despite the robust findings, the study has limitations. The cross-sectional design limits the ability to infer causality between the observed biomarkers and COVID-19 severity outcomes. Additionally, the study is confined to a single center, which may limit the generalizability of the results to other populations or ethnic groups. Moreover, the limited sample size and geographical representation of patients from a single hospital from Romania further enhanced the limitations of the study. Future studies could benefit from a multi-center approach and a longitudinal design to validate these markers across diverse populations and over time. Exploring the interaction of these biomarkers with other known risk factors could also refine their predictive accuracy and utility in clinical practice.

## 5. Conclusions

Based on the study findings, healthcare practitioners should consider implementing targeted interventions for patients with diabetes who exhibit SII values exceeding 681.742 or ALRI above 0.892 at 7 days post symptom onset, as these markers demonstrate the highest predictive capacities for severe COVID-19 outcomes. While acknowledging the study’s limitations, these insights are valuable for healthcare professionals managing COVID-19 in diabetic patients, advocating for the inclusion of these biomarkers in routine assessments to better anticipate and manage severe disease trajectories.

## Figures and Tables

**Table 1:** Background characteristics of COVID-19 patients with and without diabetes mellitus.

Variables	No Diabetes (*n* = 168)	Diabetes (*n* = 168)	Control (*n* = 54)	*p*-Value *
Age, years (mean ± SD)	50.5 ± 12.3	52.8 ± 11.7	51.1 ± 13.8	0.080
Gender, men	92 (54.76%)	98 (58.33%)	29 (53.70%)	0.439
BMI (mean ± SD)	26.8 ± 4.2	27.3 ± 5.1	28.0 ± 4.8	0.327
Smoking	34 (20.24%)	29 (17.26%)	12 (22.22%)	0.525
Alcohol use	40 (23.81%)	35 (20.83%)	15 (27.78%)	0.461
Place of origin (urban)	130 (77.38%)	125 (74.40%)	41 (75.93%)	0.497
Diabetes type	-	-	-	-
T1DM	-	30 (17.86%)	20 (37.04%)	-
T2DM	-	138 (82.14%)	34 (62.96%)	-
COVID-19 vaccinated	142 (84.52%)	150 (89.29%)	40 (74.07%)	0.204
CCI >2	50 (29.76%)	75 (44.64%)	22 (40.74%)	0.005
COVID-19 severity	-	-	-	0.429
Mild	105 (62.50%)	96 (57.14%)	-	
Moderate	47 (27.98%)	58 (34.52%)	-	
Severe	16 (9.52%)	14 (8.33%)	-	
ICU admissions	12 (7.14%)	26 (15.48%)	-	0.016
Mechanical ventilation	6 (3.57%)	19 (11.31%)	-	0.007
Mortality	4 (2.38%)	12 (7.14%)	-	0.040

SD—standard deviation; BMI—body mass index; T1DM—type 1 diabetes mellitus; T2DM—type 2 diabetes mellitus; CCI—Charlson Comorbidity Index; ICU—intensive care unit; *—data compared between no diabetes and diabetes groups.

**Table 2:** Comparison of laboratory findings at 3 days from first symptom onset between COVID-19 patients with and without diabetes.

Variables (Mean ± SD)	Normal Range	No Diabetes (*n* = 168)	Diabetes (*n* = 168)	Control (*n* = 54)	*p*-Value *
Laboratory markers					
Blood glucose (mg/dL)	70–99	97.36 ± 14.28	159.22 ± 49.73	148.75 ± 48.94	<0.001
C-reactive protein (mg/L)	<5	39.64 ± 34.15	69.97 ± 49.86	3.82 ± 1.97	0.002
D-dimer (μg/mL)	0–0.5	0.48 ± 0.32	0.68 ± 0.39	0.42 ± 0.18	0.014
Creatinine (mg/dL)	0.6–1.2	0.97 ± 0.22	1.34 ± 0.36	1.12 ± 0.26	0.003
Lymphocyte count (×10^9^/L)	1.0–3.0	1.52 ± 0.46	1.18 ± 0.43	2.15 ± 0.58	0.01
Platelets	150–400	218.73 ± 43.58	209.94 ± 49.31	288.15 ± 36.79	0.321
WBC (×10^9^/L)	4.0–10.0	6.47 ± 1.56	6.98 ± 1.83	7.55 ± 1.34	0.112
AST	10–40	29.82 ± 9.76	44.98 ± 19.52	25.98 ± 7.21	<0.001
ALT	10–50	24.87 ± 14.62	54.62 ± 24.67	22.45 ± 9.87	<0.001
Laboratory scores					
dNLR	-	2.18 ± 0.83	3.04 ± 1.18	1.98 ± 0.62	<0.001
ALRI	-	0.53 ± 0.21	1.12 ± 0.33	0.38 ± 0.15	<0.001
APRI	-	0.43 ± 0.13	1.65 ± 0.24	0.41 ± 0.11	<0.001
SII	-	510.36 ± 147.22	752.41 ± 248.36	450.36 ± 127.58	<0.001

SD—standard deviation; WBC—white blood cell; dNLR—derived Neutrophil to Lymphocyte Ratio; ALRI—ALT to Platelet Ratio Index; APRI—AST to Platelet Ratio Index; SII—Systemic Inflammation Index; *—Data compared between no diabetes and diabetes groups.

**Table 3:** Comparison of laboratory findings at 7 days from first symptom onset between COVID-19 patients with and without diabetes.

Variables (Mean ± SD)	Normal Range	No Diabetes (*n* = 168)	Diabetes (*n* = 168)	Control (*n* = 54)	*p*-Value *
Laboratory markers					
Blood glucose (mg/dL)	70–99	95.42 ± 10.67	149.53 ± 39.86	144.29 ± 47.81	<0.001
C-reactive protein (mg/L)	<5	31.74 ± 19.83	54.32 ± 30.47	3.03 ± 2.16	<0.001
D-dimer (μg/mL)	0–0.5	0.42 ± 0.21	0.63 ± 0.28	0.57 ± 0.24	0.006
Creatinine (mg/dL)	0.6–1.2	0.91 ± 0.19	1.27 ± 0.28	1.31 ± 0.24	0.001
Lymphocyte count (×10^9^/L)	1.0–3.0	3.83 ± 0.45	4.38 ± 0.48	2.41 ± 0.36	<0.001
Platelets	150–400	231.78 ± 40.35	202.14 ± 45.67	281.35 ± 61.48	0.033
WBC (×10^9^/L)	4.0–10.0	6.01 ± 1.04	6.79 ± 1.69	7.28 ± 1.09	<0.001
AST	10–40	28.94 ± 7.82	38.94 ± 7.82	32.16 ± 6.20	<0.001
ALT	10–50	19.73 ± 9.46	46.27 ± 22.89	30.31 ± 17.25	<0.001
Laboratory scores					
dNLR	-	2.02 ± 0.71	2.79 ± 1.09	1.87 ± 0.89	0.002
ALRI	-	0.42 ± 0.17	0.93 ± 0.22	0.36 ± 0.31	<0.001
APRI	-	0.47 ± 0.12	1.19 ± 0.21	0.42 ± 0.16	<0.001
SII	-	466.58 ± 121.97	702.34 ± 229.86	229.42 ± 75.96	<0.001

SD—standard deviation; WBC—white blood cell; dNLR—derived Neutrophil to Lymphocyte Ratio; ALRI—ALT to Platelet Ratio Index; APRI—AST to Platelet Ratio Index; SII—Systemic Inflammation Index; *—Data compared between no diabetes and diabetes groups.

**Table 4:** Best cutoff values for severe COVID-19 prediction in patients with diabetes.

Laboratory Parameter	Timeframe	Best Cutoff Value	Sensitivity	Specificity	AUC	*p*-Value
dNLR	At 3 days	2.685	70.312	65.978	0.624	<0.001
ALRI	At 3 days	0.812	76.429	69.541	0.752	<0.001
APRI	At 3 days	0.965	62.837	67.395	0.599	0.006
SII	At 3 days	645.285	74.158	68.264	0.768	<0.001
dNLR	At 7 days	2.539	72.105	67.321	0.652	<0.001
ALRI	At 7 days	0.892	74.991	70.002	0.756	<0.001
APRI	At 7 days	1.101	69.483	73.51	0.706	<0.001
SII	At 7 days	681.742	75.869	69.415	0.781	<0.001

dNLR—derived Neutrophil to Lymphocyte Ratio; ALRI—ALT to Platelet Ratio Index; APRI—AST to Platelet Ratio Index; SII—Systemic Inflammation Index.

**Table 5:** Cox proportional hazards regression analysis for severe COVID-19 development in patients with diabetes.

Factors above the Best Cutoff	Best Cutoff Values	Hazard Ratio	95% CI	*p*-Value
dNLR (3 days)	2.685	1.32	1.05–2.66	0.018
ALRI (3 days)	0.812	1.48	1.12–1.97	0.006
APRI (3 days)	0.965	1.25	0.99–1.58	0.057
SII (3 days)	645.285	1.53	1.20–3.92	0.001
dNLR (7 days)	2.539	1.27	1.01–1.59	0.042
ALRI (7 days)	0.892	2.01	1.12–3.78	0.003
APRI (7 days)	1.101	1.35	1.07–3.71	0.012
SII (7 days)	681.742	2.62	1.29–5.04	<0.001

dNLR—derived Neutrophil to Lymphocyte Ratio; ALRI—ALT to Platelet Ratio Index; APRI—AST to Platelet Ratio Index; SII—Systemic Inflammation Index; CI—confidence interval.

**Table 6:** Multivariate analysis of COVID-19 severity predictors.

Variable	Unadjusted HR (95% CI)	*p*-Value	Adjusted HR (95% CI)	*p*-Value	Factors
dNLR > Cutoff	1.35 (1.10–1.67)	0.005	1.22 (0.98–1.52)	0.075	Adjusted for age, BMI, CCI
ALRI > Cutoff	1.50 (1.20–1.87)	0.001	1.33 (1.05–1.68)	0.02	Adjusted for age, gender, CCI
APRI > Cutoff	1.45 (1.19–1.76)	0.001	1.25 (0.99–1.58)	0.06	Adjusted for underlying diseases
SII > Cutoff	1.60 (1.30–1.95)	<0.001	1.45 (1.15–1.83)	0.002	Adjusted for type of diabetes
Type of Diabetes (Type 2 vs. Type 1)	-	-	1.20 (0.90–1.60)	0.2	Included in multivariable model
Duration of Diabetes (per year)	-	-	1.05 (1.01–1.09)	0.01	Included in multivariable model

dNLR—derived Neutrophil to Lymphocyte Ratio; ALRI—ALT to Platelet Ratio Index; APRI—AST to Platelet Ratio Index; SII—Systemic Inflammation Index; CI—confidence interval.

## Data Availability

The data presented in this study are available on request from the corresponding author.
